# Science—an important lever to tackle sustainability in the specialty chemical industry

**DOI:** 10.1093/nsr/nwad193

**Published:** 2023-07-14

**Authors:** Wen-Juan Zhou, Pascal Metivier

**Affiliations:** Efficient Products and Processes Laboratory (E2P2L), UMI 3464 CNRS-Solvay, China; Research & Innovation, Solvay SA, Belgium

## Abstract

Achieving carbon neutrality in the chemical industry necessitates a green and efficient transformation. Working together, industry and academia hold the key to developing clean chemical processes, which is crucial.

Carbon neutrality by the mid-twenty-first century is essential in order to limit global warming to ≤1.5°C, which is also laid down in the Paris Agreement signed by 196 countries including China, the European Union and the USA, etc. [1]. The chemical industry is crucial to building a sustainable global economy to contribute to carbon neutrality. Carbon neutrality in the chemical industry requires a green and efficient transformation of the sources of energy, raw materials and structure of the sector. Not limited to the above requirement, He *et al.* point out that the transformation of CO_2_ is the key factor for developing a carbon cycle system to achieve the goal of carbon neutrality [2].

Compared with commodities, specialties are generally low-volume products with a globally low absolute impact, but can have a high relative impact when the CO_2_ emissions calculation is based on unit quality; in particular, they are generally at the end of a complex chain of successive reactions and all the steps have to be considered. Thus, it is also key to reduce the impact, particularly when technologies developed are applied to a wide range of products.

Solvay as a specialty chemicals company has established 2030 sustainability goals (called Solvay One Planet) to address key environmental and societal challenges [3]. The Solvay One Planet goals consist of 10 ambitious objectives around three pillars: protecting the climate, preserving resources and fostering better life. The objectives align with the reduction of our global impact and limiting greenhouse gas emissions consistently with the Paris Agreement; 2030 represents an important milestone in Solvay’s journey toward carbon neutrality by 2040.

In the specialty chemical industry, carbon neutrality requires a holistic approach that considers impacts along the value chain, from raw material sourcing, upstream production, downstream production to end-of-life waste management (Fig. 1). Thus, we aim to reach carbon neutrality through three key focus areas: sustainable products, green processes and circular systems. For products, we develop materials that reduce energy usage and emissions, such as lightweight materials replacing metals in automotive and materials for energy storage such as batteries and fuel cells. For processes, we minimize CO_2_ emissions and non-renewable energy consumption by using renewable energy and green production techniques with zero pollution and waste. For circularity, we utilize bio-based and recycled materials to avoid competing with the food chain; for example, recycled polymers can be used as raw materials for chemicals production. By taking a value-chain approach with these three mutually dependent and reinforcing focus areas, the specialty chemicals industry can transition fully to carbon-neutral and sustainable operations.

**Figure 1.**
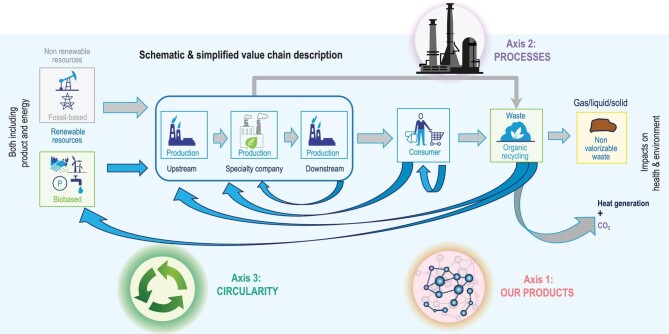
Specialty chemicals are integrated in complex value chains and the impact needs to be looked at in a holistic approach.

Using renewable raw material such as plant-based feedstocks requires a set of new performing reactions compared with the traditional reactions optimized for petrochemical feedstocks in the twentieth century. Catalysis is a key enabler of sustainability and carbon neutrality. Catalysis enables reactions that can largely eliminate side products and waste. Catalytic processes can also significantly reduce energy consumption and CO_2_ emissions. The following are two examples of how Solvay is achieving carbon neutrality through catalysis.

Example 1 is the transition from fossil to renewable resources: cyclopentanone from furfural. Cyclopentanone is an important feedstock to produce insecticides, pharmaceuticals and perfume. Currently, cyclopentanone is manufactured from petro-derived adipic acid, which generates significant carbon emissions across its multistep process (oil → benzene → cyclohexane → cyclohexanol/cyclohexanone oil → adipic acid). A promising alternative is to produce cyclopentanone from the bio-based chemicals furfural or furfuryl alcohol, especially furfural, which is derived from agricultural waste lignocellulosics. Our study demonstrated that cyclopentanone can be catalytically synthesized from furfuryl alcohol with high yield. The new catalytic route can reduce both non-biogenic CO_2_ emissions and energy usage, thereby decreasing the overall environmental impact to the conventional petrochemical route.

Example 2 involves reducing energy consumption and CO_2_ emissions in the process of phenol hydroxylation. Diphenols (hydroquinone and catechol) are two important chemicals widely used in the fine chemicals. Current industrial processes require intensive energy consumption due to low phenol conversion (5%) per step and extensive solvent/phenol recycling. Catalytic hydroxylation of phenol with H_2_O_2_ is a clean route to accessing hydroquinone and catechol, producing water as the only by-product. The development of the new Ti-UZM-35 catalyst in collaboration with East China Normal University has led to the highest results reported for phenol hydroxylation: high conversion of phenol and H_2_O_2_ with an extraordinary selectivity for hydroquinone [4]. CO_2_ reduction is ∼30 kt/y when applied to one industrial plant.

In summary, achieving sustainable industrial chemistries demands a holistic solution. For specialty chemicals produced in low volumes, key aspects requiring change may differ significantly depending on the product. Developing new renewable processes, particularly starting materials from renewable resources, requires new environmentally friendly chemical reactions. Scientific progress is essential to enable industry progress. To develop clean chemical processes that achieve carbon neutrality, it is absolutely key that industry and academia join hands together.
